# LncRNA Malat-1 From MSCs-Derived Extracellular Vesicles Suppresses Inflammation and Cartilage Degradation in Osteoarthritis

**DOI:** 10.3389/fbioe.2021.772002

**Published:** 2021-12-15

**Authors:** Chongzhi Pan, Wenzhou Huang, Qi Chen, Jiu Xu, Guoyu Yao, Bin Li, Tianlong Wu, Changchang Yin, Xigao Cheng

**Affiliations:** ^1^ Department of Orthopedics, The Second Affiliated Hospital of Nanchang University, Nanchang, China; ^2^ Institute of Orthopedics of Jiangxi Province, Nanchang, China; ^3^ Institute of Minimally Invasive Orthopedics, Nanchang University, Nanchang, China; ^4^ Second Clinical College, Nanchang University, Nanchang, China; ^5^ Jiujiang University, Key Laboratory of Medical Transformation of Jiujiang, Jiujiang, China

**Keywords:** mesenchymal stem cells, chondrocyte, osteoarthritis, inflammation, extracellular vesicles

## Abstract

**Purpose:** Extracellular Vesicles (EVs) derived from hMSCs, have the potential to alleviate cartilage damage and inflammation. We aimed to explore the effects of EVs derived from lncRNA malat‐1-overexpressing human mesenchymal stem cells (hMSCs) on chondrocytes.

**Material and Methods:** hMSCs-derived Extracellular Vesicles (hMSCs-EVs) were identified by transmission electron microscopy and western blot. We used a Sprague-Dawley (SD) rat model of CollagenaseⅡ-induced osteoarthritis (OA) as well as IL-1β-induced OA chondrocytes. Lentiviral vectors were used to overexpress lncRNA malat‐1 in hMSCs. Chondrocyte proliferation, inflammation, extracellular matrix degradation, and cell migration were measured by Edu staining, ELISA, western blot analysis, and transwell assay. Chondrocyte apoptosis was evaluated by flow cytometry, Hoechst 33342/PI Staining, and western blot. Safranine O-fast green (S-O) staining and HE staining were used to assess morphologic alterations of the rat knee joint.

**Results:** hMSCs^malat−1^-EVs decreased MMP-13, IL-6, and Caspase-3 expression in IL-1β-induced OA chondrocytes. Moreover, hMSCs^malat−1^-EVs promoted chondrocyte proliferation and migration, suppressed apoptosis, and attenuated IL-1β-induced chondrocyte injury. Our animal experiments suggested that hMSCs^malat−1^-EVs were sufficient to prevent cartilage degeneration.

**Conclusion:** Our findings show that lncRNA malat-1from hMSCs‐delivered EVs can promote chondrocyte proliferation, alleviate chondrocyte inflammation and cartilage degeneration, and enhance chondrocyte repair. Overall, hMSCs^malat−1^-EVs might be a new potential therapeutic option for patients with OA.

## Introduction

Osteoarthritis (OA) is one of the most common age-related degenerative joint diseases and public health problems worldwide, and can cause pain, disability, social and economic loss (Glyn-Jones et al., 2015). OA is characterized by the destruction of articular cartilage, synovial inflammation, osteophyte formation, and extracellular matrix (ECM) loss (Guilak et al., 2018). The molecular mechanisms underlying the occurrence and progression of OA remain unclear, and currently there are no interventions that can successfully restore the degenerated cartilage and slow down disease progression. Therefore, it is a clinical priority to seek new treatments for OA. Targeting cartilage cell regeneration, apoptosis and of extracellular matrix loss might represent new therapeutic avenues for the management and treatment of OA.

Mesenchymal stem cells (MSCs) are a type of pluripotent stem cells with self-renewal ability, have anti-inflammatory and immune roles, and have the ability of tissue repair. Bone marrow mesenchymal stem cells are used for fracture healing, cartilage repair, kidney injury, articular cartilage injury, myocardial injury and regenerative medicine due to their superior repair ability (Zhang et al., 2015; Chen et al., 2016; Li et al., 2018a; Wang et al., 2018; Yang et al., 2018; Fu et al., 2019). Existing animal experiments and clinical studies have confirmed that intra-articular injection of MSCs can effectively delay the degeneration of articular cartilage, relieve pain and improve joint function (Desando et al., 2013; Matas et al., 2019). At present, MSCs treatment still has certain limitations and side effects, such as thrombosis (Furlani et al., 2009) and abnormal ossification (Breitbach et al., 2007). Therefore, its therapeutic effect needs further research.

In recent years, EVs have gradually become the focus and entered people’s field of vision. Evs carry proteins, mRNA, and non-coding RNA (including miRNA, lncRNA, circRNA) (Tao et al., 2017; Foers et al., 2018). Many current studies have shown that EVs have a huge potential for the MSCs to treating OA (Batrakova and Kim, 2015). The research results of Lei He et show bone marrow mesenchymal stem cell-derived EVs played an important role at protecting cartilage damage and relieving knee osteoarthritis pain in a rat model of osteoarthritis (He et al., 2020). It can be seen that EVs have great potential in the treatment of OA.

LncRNA is a new non-coding RNA widely expressed in the human genome, with a well-conserved sequence and high tissue specificity (Washietl et al., 2014). Metastasis-associated lung adenocarcinoma transcript 1 (malat-1) is a novel transcript of over 8000 nucleotides which has been shown to regulate inflammation (Puthanveetil et al., 2015), promotes chondrocyte proliferation (Yang et al., 2017). LncRNA malat-1 has been extensively studied in human systemic lupus erythematosus (SLE) (Li et al., 2020a), myasthenia gravis (MG) (Felson et al., 2000), cardiovascular disease (Zhu et al., 2019), osteoporosis (Yang et al., 2019), and cancer (Fu et al., 2020). Our previous studies have also found that lncRNA malat-1 in chondrocytes promotes chondrocyte proliferation, suppresses chondrocyte apoptosis, and reduces extracellular matrix degradation (Kong et al., 2019). But to the best of our knowledge no studies have confirmed whether malat-1 in the EVs can protect chondrocytes in OA. Therefore, the specific role of lncRNA malat-1 needs further study. We hypothesized that EVs expressing lncRNA malat-1 can delay chondrocyte degeneration, promote chondrocyte repair, and have a therapeutic potential in OA, and might be a reliable therapeutic approach for OA.

## Materials and Methods

### Cell Culture and Co-Culture of Chondrocytes and EVs

Human MSCs (hMSCs) and human chondrocyte C28/I2 cells were acquired from ATCC (Manassas, United States) and BeNa Culture Collection (Beijing, China), respectively. Cells were cultured in DMED/F12 (Gibco, United States), containing 10% FBS (Gibco, United States) in a humidified incubator at 37°C and 5% CO_2_. Cells were passaged every 2–3 days.

To establish an OA cell model, C28/I2 cells at 60–70% confluency were treated with or without 10 ng/ml human IL-1β recombinant protein (Sigma, United States) for 24 h. Following establishment of the vitro OA model, chondrocytes were treated with normal medium, hMSCs-EVs (10 μg/ml), and hMSCs^malat−1^-EVs (EVs derived from lncRNA malat-1-overexpressing-hMSCs) for 24 h to investigate the effect of EVs, derived from lncRNA malat-1-overexpressing-hMSCs, on IL-1β-induced chondrocyte damage.

### Isolation and Identification of EVs

HMSCs were grown at 80–90% confluency, at which time EVs -free medium (UR51101, Umibio, Shanghai, China) was added. Following 48 h of culture, supernatant was collected. Cell culture media was centrifuged to remove cell debris and then mixed with EVs isolate kit (UR52121, Umibio, Shanghai, China). According to the manufacturer’s instructions, an initial spin was performed at 3000 ×g, at 4°C for 10 min to remove cells and cell debris. Then, the corresponding amount of reagents were added proportionally to the starting sample volume, mixed and incubated at 4°C for 2–4 h, followed by another centrifugation at 10,000 ×g for 60 min to obtain EVs pellets. Pellets were then resuspended with PBS, and purified with an EVs purification filter at 3,000 ×g for 10 min. EVs pellets were resuspended in 400 μl for 40 ml according to the manufacturer’s instructions. EVs were stored at −80°C immediately after isolation until further analysis.

BCA protein assay kit (Beyotime, China) was used to assess EVs concentration. EVs morphological appearance was observed by transmission electron microscopy (TEM, H-7700, Hitachi, Japan), and EVs surface markers were analyzed by western blot (WB).

### Uptake of PKH26‐Labelled hMSCs‐EVs

For up-take experiments, hMSCs-EVs were labeled with a red fluorescent color PKH26 kit (Sigma-Aldrich, United States), according to the manufacturer’s instructions. Briefly, hMSCs‐EVs were resuspended in 500 µl Diluent C, 2 µl PKH26 dye was then added to 500 µl Diluent C and incubated for 5 min at room temperature (RT). Then, 1 ml of 0.5% EVs‐depleted FBS was added and incubated for 5 min to allow binding to excess dye. hMSCs‐EVs were then collected by centrifugation at 100,000 ×g, at 4°C for 1 h. Next, hMSCs‐EVs were resuspended in PBS and co-cultured with chondrocyte cells. Twenty-four hours later, cells samples were fixed in 4% paraformaldehyde, stained by 4′, 6‐diamidine‐2′2phenylindole dihydrochloride (DAPI) (Beyotime, Shanghai, China) for 10 min at RT, and observed under a fluorescence microscope (Novel, China) at a magnification of ×400.

### Cell Transduction and Transfection

Lentiviral vectors targeting malat-1 were purchased from Focus Bioscience (Nanchang, China). Briefly, lentiviral vectors were transfected into competent cell 293T by using Lipofectamine 2000 (Thermo Fisher Scientific, United States). Culture medium was collected and co-cultured with hMSCs. HMSCs were infected lentivirus medium for 16 h, following which medium was replaced with fresh medium for another 48 h. Then, expression of lncRNA malat-1 was assessed by qRT-PCR. Infected hMSCs cells were co-cultured with puromycin (1 µg/ml) until all cells in the control group were dead. hMSCs overexpressing lncRNA malat-1 were filtrated and used for the next experiments.

### Cell Viability Assay

CCK-8 was used to detect the effect of EVs on IL-1β-induced-chondrocyte injury. Chondrocyte cells (2 × 10^3^) were cultured in a 96-well plate overnight, and co-cultured with different EVs concentrations (0, 1, 5, 10, 25, and 50 μg/ml) for 24 h. A control group was only co-cultured with normal culture media. According to the manufacturer’s protocol, cells were incubated with 10 μl of CCK-8 solution (TransGen, Beijing, China) in each well for 4 h at 37°C. Finally, the optical density (OD) value at 450 nm of each well was measured by using a microplate reader (Perlong, Beijing, China).

### Edu Proliferation Assay

The effects of EVs on chondrocytes were assessed with the Edu Cell Proliferation Assay Kit (Beyotime, China). Cells were seeded in 12-well plates and treated with IL-1β or EVs for 24 h. Following the manufacturer’s manual, 10 μM of Edu solution was added to the plates in an incubator at 37°C for 2 h before fixation and permeabilization. Then, cells were incubated with Click Additive Solution at RT and kept away from light for 30 min. Next, chondrocytes cell nuclei were stained with Hoechst 33342 according to the manufacturer’s instructions. The proportion of cells incorporating Edu was assessed under a fluorescence microscope.

### Transwell Assay

Transwell cell culture chamber assay was used to assess cell migration. After treatment with IL-1β or different EVs for 24 h, chondrocytes were digested and resuspended in 2 ml serum-free medium. Then count the cells to ensure that the cell concentration is 7.5 × 10^4^/ml. Next, 400 μl of cell suspension was added to the upper chamber (Corning, United States), and 700 μl DMEM containing 10% FBS was added to each bottom chamber of the 24-well plate. Following co-cultured for 24 h chondrocytes were washed twice with PBS, and fixed 4% paraformaldehyde for 20 min at RT. Then, chondrocytes were stained with 0.5% crystal violet dye for 30 min. Finally, the number of migrating cells was counted under a microscope.

### Annexin V-FITC/Propidium Iodide Flow Cytometry Assay

Chondrocytes were cultured in 6-well plates and treated with IL-1β and EVs. After 24 h incubation, cells were resuspended with PBS, cells were counted, then resuspended in binding buffer, followed by incubation with Annexin V-FITC and PI (Beyotime, China) for 20 min at RT in a dark place. The rate of apoptosis was measured by flow cytometry (BD, San Jose, CA, United States).

### Hoechst 33342/PI Assay

Hoechst 33342/PI staining kit was acquired from Solarbio (Beijing, China). According to the manufacturer’s instructions, dye and cell staining buffers were added to each well incubated at 4°C for 30 min in a dark place after cell treatment with IL-1β, hMSCs-EVs, or hMSCs^malat−1^-EVs. Finally, cells were observed with a fluorescence microscope.

### IL-6 ELISA Assay

Following culture and treatment of chondrocyte cells with IL-1β or EVs for 24 h, the supernatants of each group were collected. The concentration of IL-6 was measured with a specific ELISA kit (Neobioscience, Shenzhen, China) following the manufacturer’s instructions. In brief, 100 μl of the control, standard, or sample was added to each well and incubated for 90 min at 37°C. Then, wells were washed five times and incubated with 100 μl of human IL-6 conjugate antibody for 1 h at 37°C and washed again five times. 100 μl of the enzyme combination solution was added into each well and incubated for 30 min at 37°C away from light, followed by another step of washes. 100 μl of substrate solution was then added to each well and incubated for 15 min at 37°C away from the light. Finally, 100 μl of Stop Solution was added, and the OD values were measured at 450 nm by using a microplate reader.

### Quantitative Real-Time Polymerase Chain Reaction

Total RNA was isolated from cells using Trizol reagent (Takara, Japan). Reverse transcription was carried out with the PrimeScrip RT reagent Kit with gDNA Eraser (Takara, Japan). TB Green Premix Ex Taq kit (Takara, Japan) was used to perform real-time PCR, and we used the CFX96 Real-Time PCR Detection System (Bio-Rad, United States) with the following primers: HMSC GAPDH: forward, 5′-GGT​GGT​CTC​CTC​TGA​CTT​CAA​CA-3′ and reverse, 5′-TTG​CTG​TAG​CCA​AAT​TCG​TTG​T-3′; HMSC malat-1:forward, 5′- TCA​GGA​TAA​TCA​GAC​CAC​CAC​AG-3′ and reverse, 5′- GTA​ACT​ACC​AGC​CAT​TTC​TCC​AA-3′; Internal control was GAPDH.

### Western Blot

Cells were washed three times with PBS and lysed in RIPA buffer with a protease inhibitor cocktail at the ice temperature for 20 min. After centrifugation at 10,000 ×g and 4°C for 10 min, the concentration of total protein levels was measured using the BCA kit. Protein extracts were separated by polyacrylamide gel electrophoresis (12–15%) and transferred to polyvinylidene difluoride (PVDF) membranes. Then, blots were incubated overnight at 4°C with antibodies specific for GAPDH (1:5000,Abcam,United States), CD63 (1:1000, Proteintech, China), CD81 (1:1000,Abcam, United States), TSG101 (1:1000,Abcam, United States), caspase-3 (1:5000,Abcam, United States), IL-6 (1:3000,Proteintech, China),MMP-13 (1:500, Proteintech, China). After washing with TBST 3 times, membranes were incubated with a goat anti-rabbit IgG–horseradish peroxidase antibody. Proteins were visualized using the ECL Chemiluminescent Kit (UE, Suzhou, China), and the chemical luminescence reaction was detected by the TECAN luminescent imaging system.

### Animal Studies

All animal experiments were approved by the Institutional Animal Care and Use Committee of Nanchang University (China) and followed by the Guide for the Care and Use of Laboratory Animals by the National Institutes of Health (NIH). 10-week-old male Sprague-Dawley (SD rats) were obtained from the Experimental Animal Center of Nanchang University (Nanchang, China). Animals were kept in an environment with a dark/light cycle at 20–25°C, at ad libitum access to water and food. Animals were randomly divided into four groups randomly (n = 4 per group): Normal; OA + PBS; OA + hMSCs-EVs; OA + hMSCs^malat−1^-EVs. To establish the OA model, collagenase Ⅱ was injected into the knee joint cavity of SD rats under anesthesia. Three weeks after the injection, hMSCs-derived EVs were injected into the articular cavity of rats once a week at a concentration of 40 μg/100 μl. Six weeks later, histologic analysis was performed on the knee joint specimens.

### Haematoxylin and Eosin and Safranine O-Fast Green Staining

Six weeks after treatment with EVs, all animals were sacrificed and articular cartilage samples were collected, fixed in paraformaldehyde for 24 h and subject to calcium removal for 21 days in 10% EDTA (pH 7.4). Then, knee-joint tissues were embedded in paraffin and sectioned into 5-μm-thick sections. Serial sections were obtained from the medial and lateral compartments at 200-μm intervals. Selected sections were deparaffinized in xylene, rehydrated through a graded series of ethanol washes, and stained by hematoxylin and eosin (H&E) and Safranin O/Fast Green staining (Solarbio, China). The degree of articulatio genus cartilage degeneration on the medial and lateral tibial plateau joint was evaluated according to the Osteoarthritis Research Society International (OARSI) score and the modified Mankin’s score.

### Statistical Analysis

All experiments were performed in triplicate. Mean ± standard deviation (SD) was used to present the data. Graphics and statistical analyses were conducted by GraphPad Prism 8 software (GraphPad Inc., La Jolla, CA, United States). Student’s t-test for two groups and one-way ANOVA with Tukey post hoc test for three or more groups were used for group comparisons. A *p* value of less than 0.05 was considered statistically significant.

## Results

### Identification of EVs and Isolated From Human BMSCs

**FIGURE 1:**
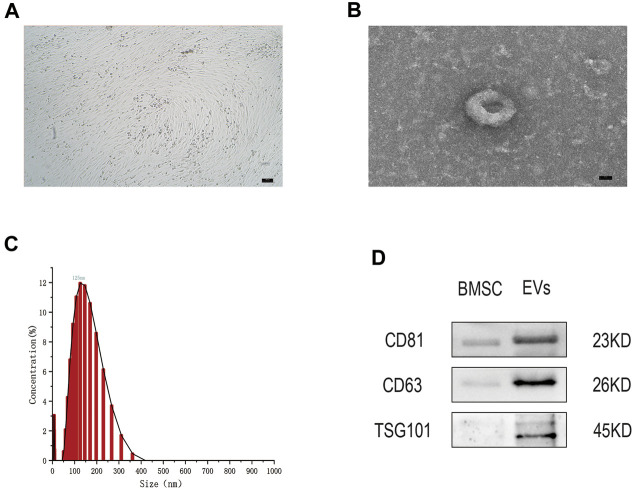
**(A)** The hMSCs were observed by optical microscope. Scale bar = 100 μm. **(B)** The cup-shaped EVs under TEM. Scale bar = 25 nm **(C)** Size and concentration of EVs analyzed by NTA. **(D)** The indicated protein (CD9, CD63, and TSG101) in EVs were detected *via* western blotting. *n* = 3.

After 48 h in culture, bone marrow mesenchymal stem cells (hMSCs) showed a uniform spindle morphology (Figure 1A). When the cell confluency reached 80%, EVs were extracted and identified by TEM, NTA, and WB. TEM results showed that EVs exhibited a classic approximate circular cup-shaped structure (Figure 1B). NTA showed a main peak of the particle size of approximately 144 nm (Figure 1C). Furthermore, WB showed that surface molecular markers CD63, CD81, TSG101 of the EVs were highly expressed compared to those of the negative group PBS (Figure 1D, Supplementary Data Sheet S2). These results show that EVs were successfully separated from the hMSCs.

### Establishment of OA Model of Chondrocytes and EVs Uptake Experiment

**FIGURE 2:**
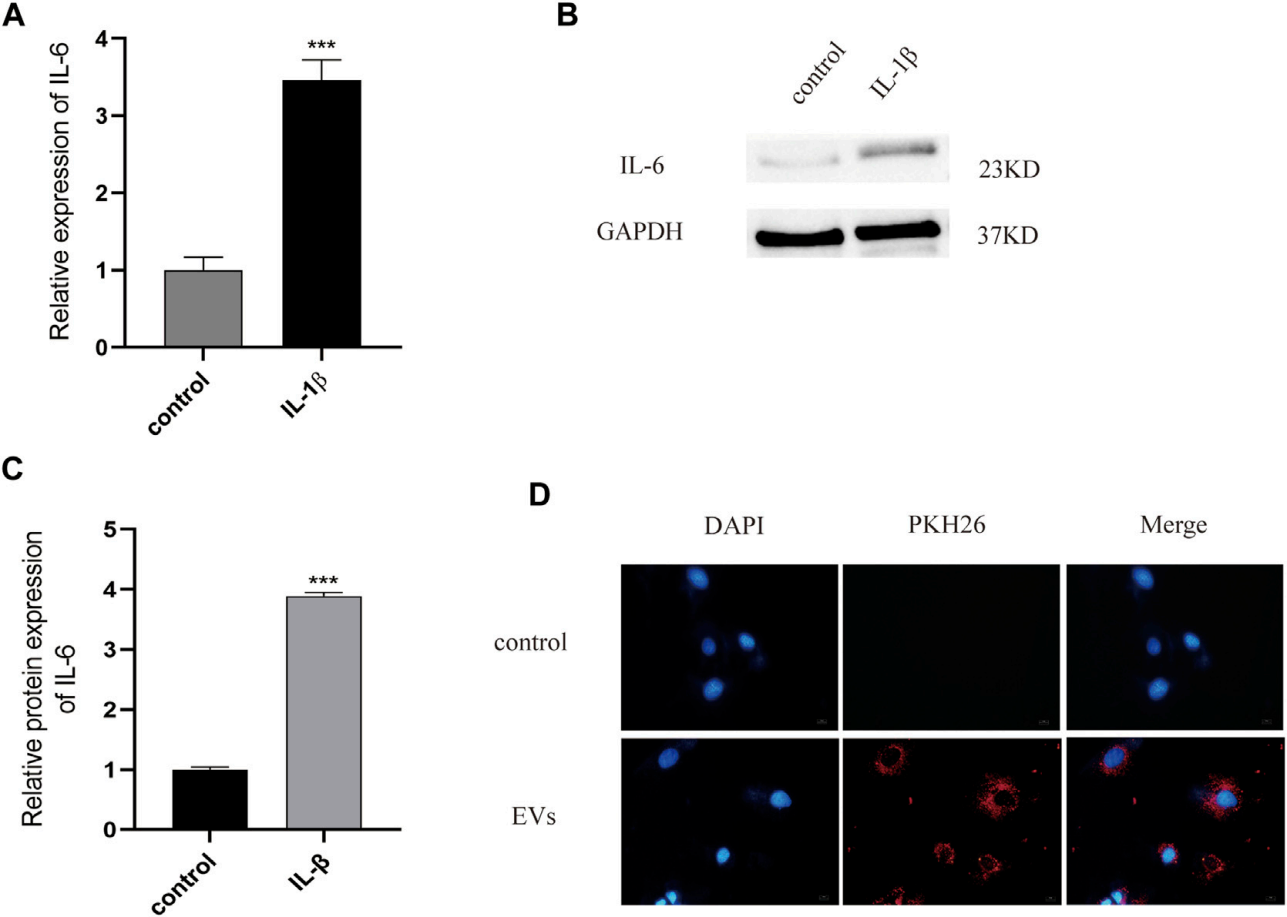
**(A)** The expression of inflammatory factor IL-6 analyzed by Elisa. **(B)** and **(C)** The protein of IL-6 measured by western blot. **(D)** PKH26-labeled were uptaked by chondrocyte. Scale bar = 10 μm, *n* = 3, ***p < 0.001, t-test for all.

According to previous literature, IL-1β (10 ng/ml) is appropriate for establishing an inflammatory injury model of chondrocytes to simulate the microenvironment of OA. ELISA experiments have shown that, after IL-1β treatment, the expression of the inflammatory cytokine IL-6 in chondrocytes was significantly higher than in the control group (*p* < 0.05) (Figure 2A). The expression of this inflammatory factors was also detected by western blot, with the results showing that IL-6 protein expression in the OA model group was increased by 3.89 times (*p* < 0.05) (Figures 2B,C, Supplementary Data Sheet S1). All data were statistically significant. These results indicate that the inflammatory injury model of chondrocytes has been successfully established.

To test the potential of EVs in the treatment of OA, we used PKH26 labeled EVs (red fluorescence), which were then co-cultured with chondrocytes. After 24 h of co-culture, EVs labeled with PKH26 were successfully observed in the cytoplasm of chondrocytes by fluorescence microscopy, suggesting that the EVs had been taken up by chondrocytes (Figure 2D).

### LncRNA Malat-1 Overexpression in the EVs Reduces IL-1β Induced Inhibition of Chondrocyte Proliferation

**FIGURE 3:**
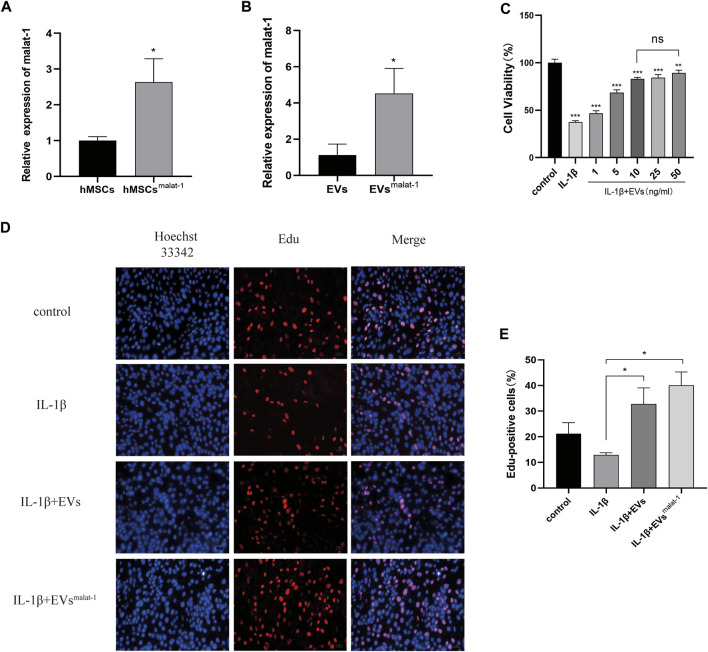
**(A)** The expression of lncRNA malat-1 were analyze between hMSCs and hMSCs^over-malat−1^ by qRT-PCR, *n* = 3, *p < 0.05, t-test. **(B)** The expression of lncRNA malat-1 in two EVs analyzed by qRT-PCR, n = 3, *p < 0.05, t-test. **(C)** Cell viability of chondrocytes treated with different concentrations of EVs by CCK-8 assay. *n* = 3, *p < 0.05, one-way ANOVA **(D)** and **(E)** Edu assay was performed to detect proliferation of chondrocytes, *n* = 3, *p < 0.05, one-way ANOVA.

To obtain lncRNA malat-1-rich EVs, lentiviral vectors were used to overexpress malat-1 in hMSCs. qRT-PCR data showed that malat-1 expression increased by approximately 2.6 times compared to the control group (*p* < 0.05) (Figure 3A). EVs were then extracted from the hMSCs cell serum, and lncRNA malat-1 expression in hMSCsmalat-1-EVs, detected by qRT-PCR, was about 4.03 times higher than in hMSCs-EVs (*p* < 0.05) (Figure 3B), indicating that lncRNA malat-1 was successfully expressed in EVs. To investigate the role of EVs in OA, we investigated the effects of different concentrations of EVs on chondrocyte vitality after IL-1β treatment. We found chondrocyte vitality was significantly decreased after IL-1β treatment (*p* < 0.05). There was no significant difference in the 10 µg/ml groups compared to 25 µg/ml and 50 µg/ml concentrations (*p* > 0.05) (Figure 3C). Therefore, the concentration of 10 µg/ml was selected for subsequent experiments.

Then, the effects of hMSCs-EVs and hMSCs^malat−1^-EVs on chondrocyte proliferation were examined in the different groups. Edu staining confirmed that chondrocytes from the hMSCs^malat−1^-EVs group showed better proliferation promotion compared to the hMSCs-EVs group (*p* < 0.05) (Figures 3D,E).

### hMSCs^malat−1^-EVs Inhibited Apoptosis and Inflammation of Chondrocytes Induced by IL-1β

**FIGURE 4:**
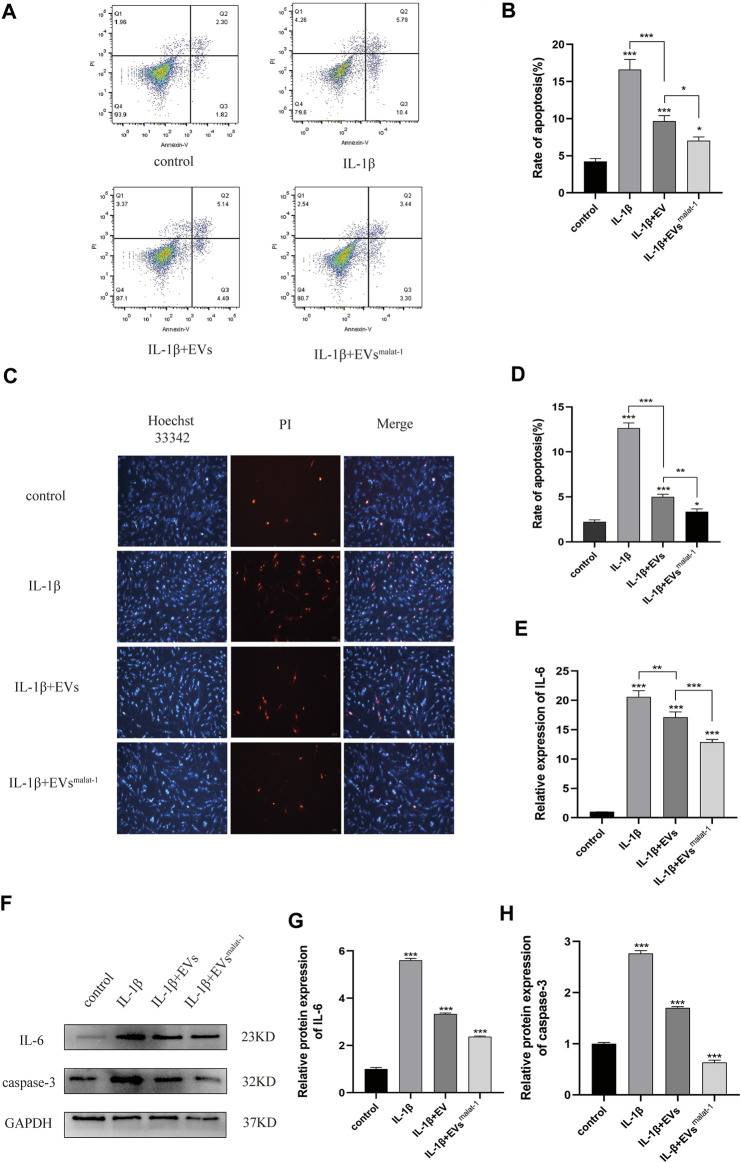
**(A)** and **(B)** The apoptosis rate of chondrocytes treated with EVs analyzed by flow cytometry assay. **(C)** and **(D)** Hoechst 33342/PI assay was used to analyze the apoptosis rate of chondrocytes. **(E)** Inflammatory factor IL-6 expression was measured using Elisa in chondrocytes after treated with EVs. **(F–H)** The protein expression of IL-6 and Caspase-3 analyzed by western blot. *p < 0.05, **p < 0.01, ***p < 0.001, vs. control group. *n* = 3, one-way ANOVA for all.

We performed additional experiments to further explore the effect of LncRNA malat-1 in EVs on IL -1β induced chondrocyte apoptosis. We measured the apoptosis rate by flow cytometry. As the result show (Figures 4A,B), the apoptosis rate of the IL-1β group (16.62 ± 1.12%, *p* < 0.05) was significantly higher than that from the normal group (4.25 ± 0.32%, *p* < 0.05). After treatment with the hMSCs-EVs, the apoptosis rate of chondrocytes (9.67 ± 0.6%, *p* < 0.05) decreased by a third. At the same time, we found that the apoptosis rate of hMSCsmalat-1-EVs group (7.02 ± 0.42%, *p* < 0.05) decreased more substantially and was closer to the normal rate. Similarly, Hoechst 33342/PI staining results showed that after IL-1β treatment, the number of apoptotic cells increased significantly (Figures 4C,D). After adding hMSCs-EVs, the number of apoptotic cells decreased, but after adding hMSCs^malat−1^-EVs, the number of apoptotic cells decreased even more substantially, suggesting a stronger anti-apoptotic effect of hMSCs^malat−1^-EVs. Caspase-3 protein expression was detected by WB, and the results were consistent with those from flow cytometry and Hoechst 33342/PI staining.

In addition, the effect of LncRNA malat - 1 in EVs on IL - 1β induced chondrocyte inflammatory injury. Based on results from ELISA and WB (Figures 4E,H, Supplementary Data Sheet S1), chondrocyte in the normal group appeared to produce only a small amount of IL - 6 inflammatory-related factors and the OA inflammatory damage was significantly higher (*p* < 0.05). following EVs treatment, expression levels of IL-6 inflammation-related factors decreased (*p* < 0.05), but compared with the hMSCs-EVs group, the decrease in the hMSCs^malat−1^-EVs group was more substantial (*p* < 0.05). These results suggest that chondrocyte apoptosis and inflammation induced by IL-1β can be inhibited by lncRNA malat-1 of the EVs, which appears protective for chondrocytes of OA.

### hMSCs^malat−1^-EVs Reversed IL-1β -Induced Decline of Chondrocyte Migration and Reeducation of MMP-13

**FIGURE 5:**
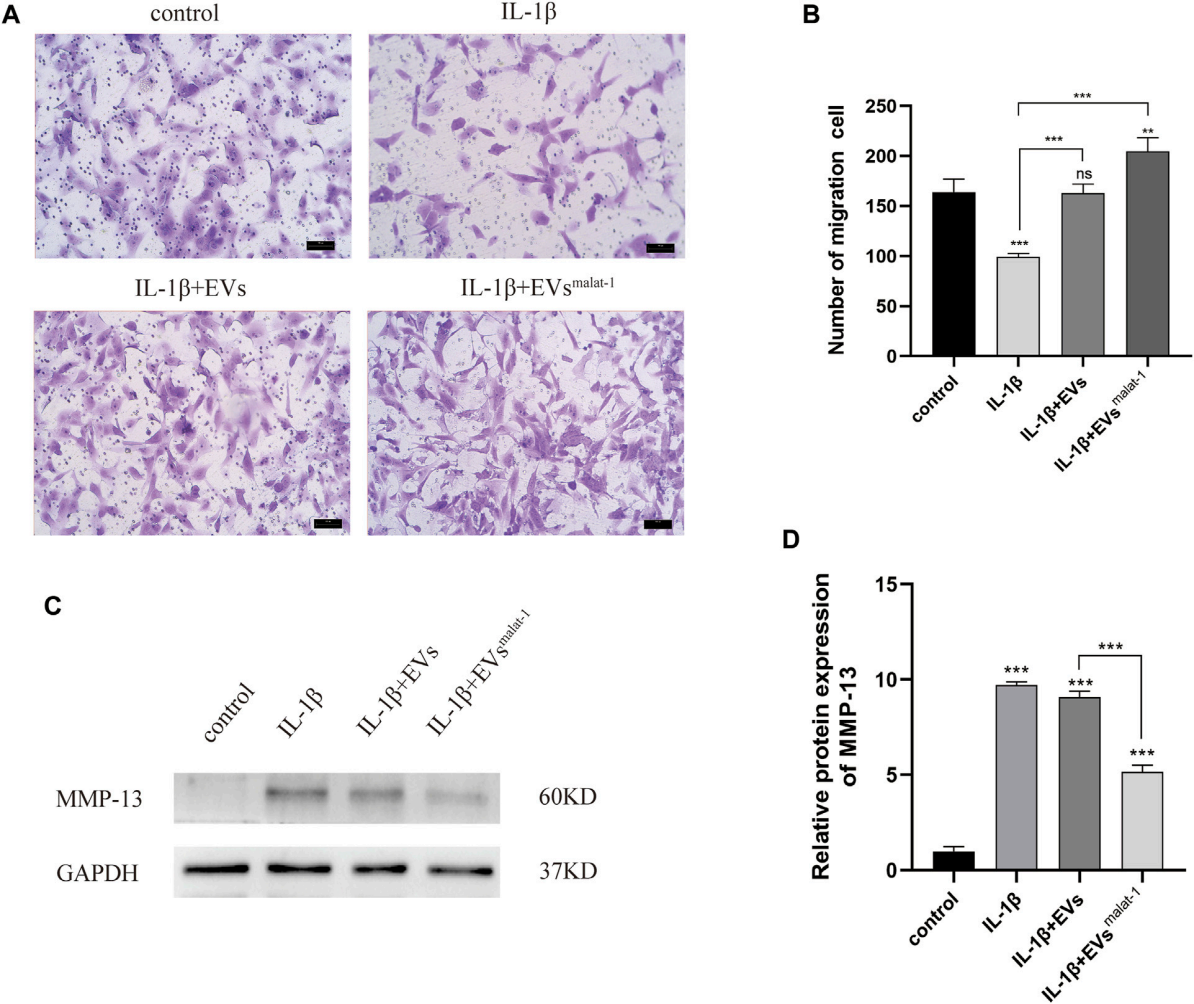
**(A)** and **(B)** Migration of chondrocytes was detected by Transwell assays. **p < 0.01, ***p < 0.001 vs. IL-1β group. **(C)** and **(D)** The protein expression of MMP-13 was detected by western blot, *p < 0.05, **p < 0.01, ***p < 0.001, vs. control group. *n* = 3, one-way ANOVA for all.

We then performed transwell experiments to test the effects of hMSCs-EVs and hMSCs^malat−1^-EVs on the ability of chondrocytes to migrate in OA. Our results showed that the number of migrating cells of IL-1β group decreased significantly compared to control groups (*p* < 0.05) (Figures 5A,B), while no significant differences were observed in the hMSCs-EVs group (*p* > 0.05). All groups, when treated with hMSCs^malat−1^-EVs, exhibited a larger number of migrating cells compared to the IL-1β group and the hMSCs-EVs group (*p* < 0.05). The results from these experiments showed that IL-1β reduced chondrocyte migration ability, while the EVs reversed these effects *via* lncRNA malat-1.

To investigate the effect of EVs by malat-1 on the extracellular matrix (ECM) in OA, matrix metalloproteinase 13 (MMP-13) protein expression was tested. We found that both EVs downregulated the increased MMP-13 protein levels induced by IL-1β (*p* < 0.05) (Figures 5C,D, Supplementary Data Sheet S1). A more substantial decrease in MMP-13 protein levels was observed in the hMSCs^malat−1^-EVs group compared to the hMSCs-EVs group (*p* < 0.05), suggesting that the EVs delayed IL-1β -induced chondrocyte degeneration through lncRNA malat-1.

### hMSCs^malat−1^-EVs Alleviated Cartilage Damage in a Rat OA Model

**FIGURE 6:**
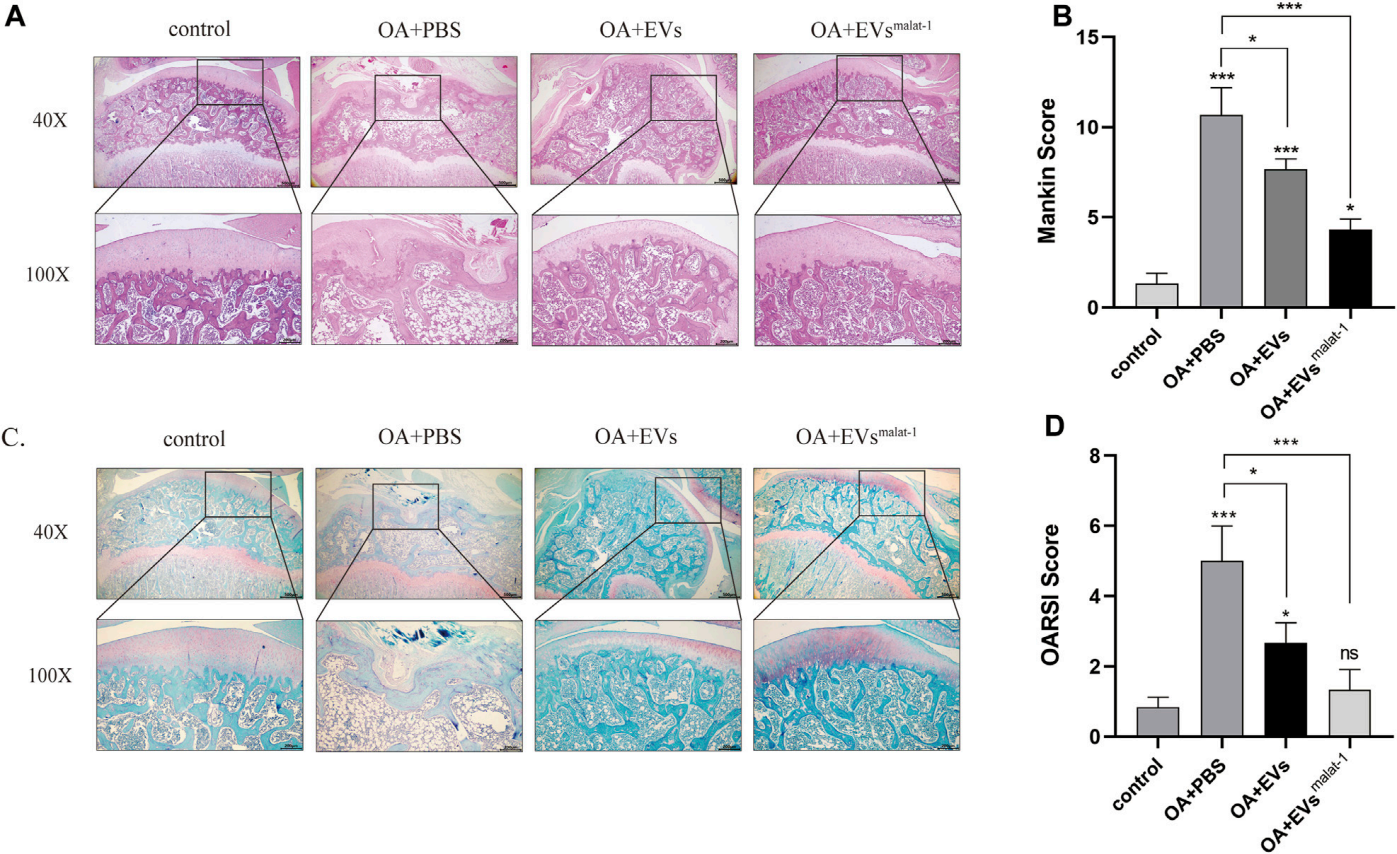
**(A)** Haematoxylin and Eosin staining of SD rat knee joints after injecting EVs for 6 weeks. Scale bar: 200 μm (40X) and 500 μm (100X). **(B)** The modified Mankin’s score of the SD rat knee joints. **(C)** Safranin O/Fast Green staining of SD rat knee joints after injecting EVs for 6 weeks. Scale bar: 200 μm (40X) and 500 μm (100X). **(D)** OARSI scores of the SD rat knee joints. *p < 0.05, **p < 0.01, ***p < 0.001, vs. control group. *n* = 3, one-way ANOVA for all.

To confirm the protective effect of hMSCs^malat−1^-EVs on chondrocytes *in vivo*, hMSCs-EVs and hMSCs^malat−1^-EVs were injected into the articular cavity of OA rats. Six weeks later, OA articular surface changes were observed by HE staining and Safranine O-fast green (S-O) staining. The articular cartilage structure of OA rats was clear and exhibited a smooth surface. In the OA group, the surface was rough and had an irregular shape, the fibers were broken, and the cartilage was substantially damaged (Figures 6A,B). The score of OARSI and Mankin (*p* < 0.05) increased significantly (Figures 6B,C). After hMSCs-EVs treatment, articular cartilage injury of OA rats was alleviated and OARSI scores and Mankin score both decreases. Compared with hMSCs-EVs, the joint surface of the hMSCs^malat−1^-EVs group was smoother, the cartilage damage was substantially alleviated, and the scores decreased (*p* < 0.05). These results indicate that hMSCs-EVs can alleviate cartilage damage caused by OA *via* LncRNA malat-1 in a rat model of OA.

## Discussion

The causes of OA are complex and multifactorial, and involve age, gender, degree of obesity, aging, genetic factors, joint damage, and other factors (Felson et al., 2000). In this study, we investigated a new potential treatment for OA. We successfully isolated EVs from hMSCs and used them to treat chondrocytes induced by IL-1β. After that, we harvested lncRNA malat-1-rich EVs through transfecting hMSCs to stably express lncRNA malat-1, which were used in subsequent experiments for evaluating their therapeutic effect on IL-1β induced-cartilage injury. Our results showed that overexpression of lncRNA malat-1 of EVs significantly reduced expression of IL-6, caspase-3, MMP-13 in OA, increased apoptosis, reversed IL-1β induced inhibition of proliferation, and increased the proliferation and migration rates of chondrocytes.

EVs are a type of vesicular structure of 40–160 nm in diameter that is actively secreted by almost all cells (Kalluri and LeBleu, 2020). EVs contain signaling factors such as mRNA, miRNA, and lncRNA, targeting adjacent cells by autocrine and paracrine mechanisms, or distal cells through the circulatory system (Colombo et al., 2014). Compared with stem cell transplantation, EVs became a hot topic given their potential for the treatment of OA due to their characteristics such as immunogenicity, and ease to save. Previous studies have suggested their huge therapeutic potential in hMSCs derived EVs in joint disease (Li et al., 2018b), and the importance of hMSCs paracrine in various diseases (Linero and Chaparro, 2014). In this study, the results of TEM, NTA, and WB indicate that we successfully extracted MSCs-derived EVs Furthermore, we confirmed that cartilage cells successfully uptake EVs by using the PKH26 probe, indicating that EVs can play a role in chondrocytes. EVs have attracted the attention of many scientists as a good carrier for treating the diseases, such as Qing Mao has researched that lncRNA KLF3-AS1 in human mesenchymal stem cell-derived EVs ameliorates pyroptosis of cardiomyocytes and myocardial infarction through miR-138-5p/Sirt1 axis (Mao et al., 2019). Huaxia Yang et also has found that lncRNA malat-1 is a novel inflammatory regulator in human systemic lupus erythematosus (SLE), and has great potential in treating inflammation (Li et al., 2020b). LncRNA malat-1 is a novel transcript of over 8000 nucleotides, and one of the most widely studied lncRNAs. It was first discovered in transcript screening related to non-small cell lung cancer (NSCLC) metastasis and patient survival (Hutchinson et al., 2007). A study of Tony Gutschner shows that lncRNA malat-1 is a critical regulator of the metastasis phenotype of lung cancer cells (Gutschner et al., 2013). In recent years, Jongchan Kim et found that malat-1 can inhibit the metastasis of breast cancer. In other diseases, lncRNA malat-1 is also widely studied, such as Huaxia Yang et has shown stem cell-derived EVs prevent aging-induced cardiac dysfunction through a novel EVs/lncRNA malat-1/NF-κB/TNF-α signaling pathway (Zhu et al., 2019). Furthermore, Xucheng Yang confirmed that lncRNA malat-1 shuttled by EVs derived from bone marrow mesenchymal stem cells-secreted EVs alleviates osteoporosis through mediating microRNA-34c/SATB2 axis (Yang et al., 2019). The exciting thing is that Hongxi Li et found that lncRNA malat-1 can regulate ECM catabolism, inflammation, apoptosis and proliferation in chondrocyte (Li et al., 2020a). Our study show lncRNA malat-1 from EVs reduced expression of IL-6, caspase-3, MMP-13 in OA, decrease apoptosis, promote proliferation and migration. It shows that lncRNA malat-1 from EVs has the ability to delay the degeneration of chondrocytes.

Inflammation plays an important role in the pathogenesis of OA, in which the chondrocytes, synovial membrane, and surrounding tissues produce large amounts of inflammatory factors such as IL-1β, IL-6, TNF-α, that accelerate the progression of osteoarthritis (Eymard et al., 2014; Li et al., 2020a). The up-regulation of inflammatory factors IL-1β, IL-6, TNF-α and other catabolic factors, such as MMP-13, can accelerate apoptosis and matrix degradation of chondrocytes. Inhibition of inflammation is therefore an important part of preventing OA development. In our study, we have detected through both ELISA and WB experiments that malat1 reduces the expression of the inflammatory factor IL-6 in the IL-1β induced OA cell model through EVs. Minqiu et al. found that SFC-miR-126-3p-Exos inhibited apoptosis and inflammation of articular cartilage and chondrocyte degradation in an SD rat model of OA (Zhou et al., 2021). In another study, Shipin Zhang et also researchers and finds that MSC EVs can effectively alleviate temporomandibular joint osteoarthritis by attenuating inflammation and restoring matrix homeostasis. Jiaping Wu et al. found reported lncRNA malat-1 inhibited inflammation of microglia through the miR-154-5p/AQP9 axis (Wu et al., 2020). In this study, we examined the effect of hMSCs^malat−1^-EVs on OA inflammation and found that hMSCs^malat−1^-EVs can reduce IL-6 levels in the OA model, in agreement with the results of Jiyong Zhang et al. (Zhang et al., 2020) Ruizhang et al. found that lncRNA malat-1 in EVs promoted proliferation and migration of non-small cell lung cancer cells (Zhang et al., 2017). In our study, chondrocyte proliferation decreased significantly, and increased apoptosis and decreased migration capacity were observed, after IL-1β treatment. After treatment with hMSCs-EVs and hMSCs^malat−1^-EVs, the negative effect of IL-1β was ameliorated, and the function and vitality of chondrocytes were restored. Moreover, comparison of the two EVs revealed that hMSCs^malat−1^-EVs had a better therapeutic effect, which was consistent with our original hypothesis. These results indicated that EVs promoted chondrocyte proliferation, inhibited apoptosis, and enhanced the migration ability of chondrocytes *via* lncRNA malat-1.

Inflammatory cytokines, such as IL-1β, IL-6, TNF-α, and others, promote the production of MMPs and aggravate the progression of OA (Goldring and Otero, 2011). MMPs are involved in the degradation of various proteins in the ECM. MMP-13 is an important member of the MMPs family, which plays an important role in degrading collagen type II in OA articular cartilage and bones, and is highly expressed in OA patients but barely expressed in healthy people (Burrage et al., 2006). MMP-13 is the main key enzyme of cartilage degeneration and can degrade the ECM. MMP-13 is considered as one of the major contributing factors in the onset of OA (Wan et al., 2020), hence inhibiting MMP-13 expression could represent a new strategy to prevent OA. Xue Chen and others found that EVs secreted by MSCs inhibited the expression of MMP-13 in the traumatic arthritis model by microRNA-136-5p targeting ELF3 (Chen et al., 2011). In the inflammatory injury model of OA generated in this work, we also found that chondrocytes highly expressed MMP-13 after IL-1β treatment, and the effect of hMSCs^malat−1^-EVs on MMP-13 was further explored. We found that both hMSCs-EVs and hMSCs^malat−1^-EVs downregulated IL-1β-induced MMP-13 increase, but hMSCs^malat−1^-EVs appeared to have a stronger protective effect. This result is consistent with our hypothesis, suggesting that hMSCs^malat−1^-EVs provide better protection to the ECM and might be a more promising treatment of OA than hMSCs-EVs. Similarly, our animal experiments showed that hMSCs^malat−1^-EVs significantly delayed the degeneration of the articular cartilage in SD rats. In this study, we did not investigate the protein changes following hMSCs^malat−1^-EVs uptake by chondrocytes, which is a topic that will be investigated in future studies.

## Conclusion

All together, our results based on an IL-1β induced OA model, indicate that EVs derived from hMSCs with lncRNA malat-1 overexpression promote chondrocyte proliferation and migration, suppress inflammation, degradation of the ECM, and appear to have a good protective effect in OA, providing a new potential therapeutic option for the prevention and treatment of OA.

## Data Availability

The raw data supporting the conclusions of this article will be made available by the authors, without undue reservation.
