# Supplementary figures and images for: LncRNA Malat-1 From MSCs-Derived Extracellular Vesicles Suppresses Inflammation and Cartilage Degradation in Osteoarthritis
Source: Front Bioeng Biotechnol. 2021 Dec 15;9:772002. doi: 10.3389/fbioe.2021.772002 (PMC8715093; doi:10.3389/fbioe.2021.772002)

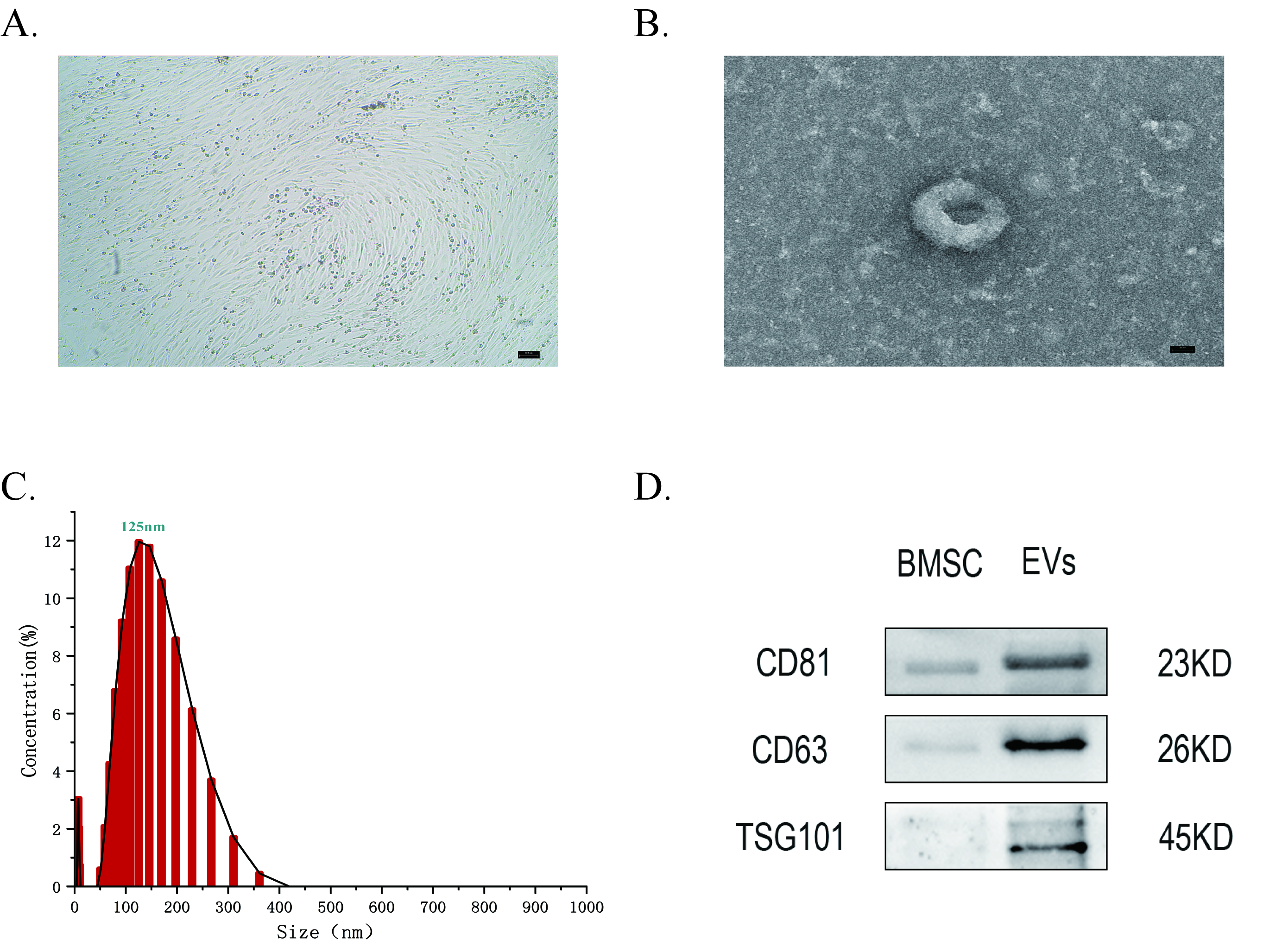

Supplement: Supplementary file 1 [file DataSheet1.zip › figure 1.tif]

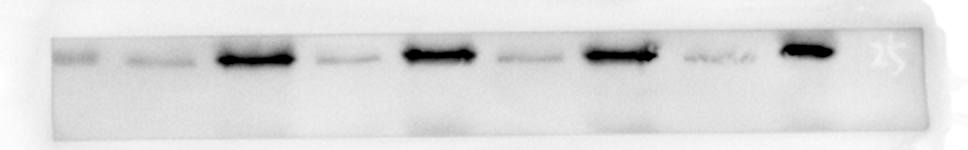

Supplement: Supplementary file 2 [file DataSheet2.ZIP › WB of figure1D/cd63.tif]

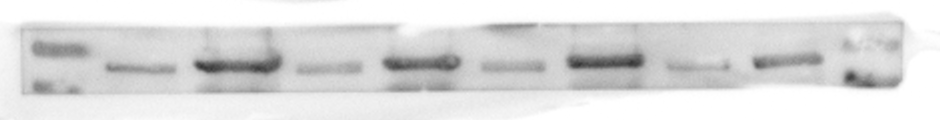

Supplement: Supplementary file 2 [file DataSheet2.ZIP › WB of figure1D/cd81.tif]

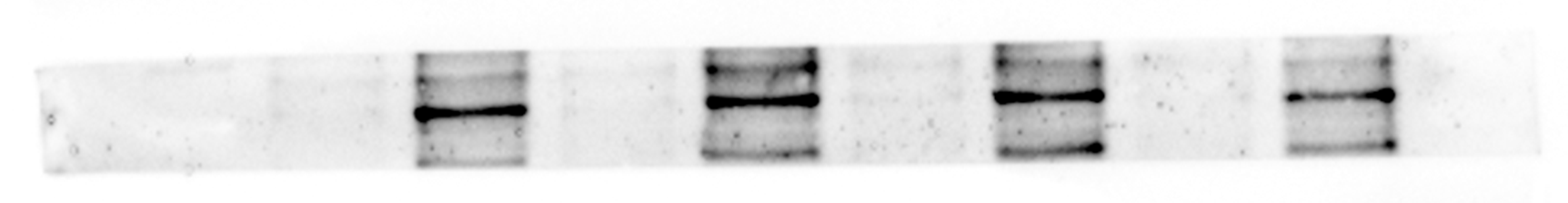

Supplement: Supplementary file 2 [file DataSheet2.ZIP › WB of figure1D/tsg101.tif]
